# Cytokeratins 18 and 8 are poor prognostic markers in patients with squamous cell carcinoma of the oesophagus

**DOI:** 10.1038/sj.bjc.6605313

**Published:** 2009-09-15

**Authors:** T Makino, M Yamasaki, A Takeno, M Shirakawa, H Miyata, S Takiguchi, K Nakajima, Y Fujiwara, T Nishida, N Matsuura, M Mori, Y Doki

**Affiliations:** 1 Department of Gastroenterological Surgery, Graduate School of Medicine, Osaka University, 2-2-E2, Yamada-oka, Suita, Osaka 565-0871, Japan; 2 Department of Molecular Pathology, School of Allied Health Science, Faculty of Medicine, Osaka University, 2-2-E2, Yamada-oka, Suita, Osaka 565-0871, Japan

**Keywords:** cytokeratin18 (CK18), cytokeratin8 (CK8), oesophageal squamous cell carcinoma (OSCC), immunohistochemistry (IHC), quantitative reverse transcription polymerase chain reaction (RT–PCR)

## Abstract

**Background:**

Cytokeratins (CKs) are structural marker proteins specific for epithelial cells. However, recent studies indicate their involvement in cancer progression.

**Methods:**

We evaluated CK18 and its filament partner, CK8 expression, by immunohistochemistry in 210 resected specimens from patients with oesophageal squamous cell carcinoma (OSCC). We also analysed the relationship between their expression and various clinicopathological parameters including prognosis.

**Results:**

Neither CK18 nor CK8 was expressed in non-cancerous squamous epithelium whereas proper oesophageal glands expressed both CKs. Ninety (42.9%) tumours were CK18 positive and 85 (40.5%) CK8 positive, and the concordance rate for immunohistochemical classification for CK18 and CK8 was 82.4%. CK18 expression correlated with poorly differentiated tumours, use of neo-adjuvant chemotherapy, and advanced stage. Prognosis of patients with CK18-positive tumours was poorer than that of patients with negative OSCC (*P*<0.001). A similar trend was noted for CK8 expression. Multivariate analysis identified pT (*P*=0.020), pN number (*P*=0.001), and CK18 expression (*P*=0.004) as independent prognostic factors. CK18 expression in 83 pretreatment biopsy specimens was detected in 47 cases (56.6%) and also correlated with prognosis (*P*=0.045).

**Conclusion:**

CK18/CK8 expression correlated with progression of OSCC. The significant correlation with prognosis and stable expression in biopsy specimen suggest usefulness of CK18 in selection of treatment strategies for OSCC.

Oesophageal squamous cell carcinoma (OSCC), the major histopathological form in East Asian countries, is one of the most lethal malignancies of the digestive tract and frequently diagnosed at advanced stage (Shimada *et al*, 2003). Although surgical resection is one of the most reliable treatments in OSCC, disease recurrence often occurs even after curative resection. Earlier reports suggested the usefulness of preoperative chemotherapy/chemoradiotherapy and postoperative chemotherapy in OSCC (Fiorica *et al*, 2004; Ando *et al*, 2003; Kaklamanos *et al*, 2003; Tepper *et al*, 2008). For effective indication of these therapeutic modalities, it is necessary to identify those patients with poor prognosis who are best candidates for these therapies. So far, pathological findings based on examination of resected specimen are the most reliable information for prediction of clinical outcome, however, the predictive values of these variables remain unsatisfactory and not available for preoperative setting. Therefore, there is a need for the development of novel biological markers that can accurately distinguish high-risk population of recurrent disease by combining conventional TNM classification (Sobin, 2002), for more appropriate treatment strategy.

Cytokeratin (CK), an intermediate filament observed mainly in epithelial cells, is an essential cytoskeletal component involved in fixation of the nucleus and maintenance of cell morphology. The cytoskeleton of epithelia is formed by 20 subtypes of CKs whose expression depends primarily on epithelial cell type and degree of differentiation (Chu and Weiss, 2002a). These CKs are divided into two groups; type I (acidic, CK9–20) and type II (neutral–basic, CK1–8) gene families (Moll *et al*, 1982; Hesse *et al*, 2001). In non-cancerous tissue, CK18 and its co-expressed complementary subunit, CK8, are commonly expressed in normal glandular epithelia, transitional cell epithelium, and hepatocyte, but not in squamous stratified epithelium (Debus *et al*, 1984; Schaafsma *et al*, 1990; Trask *et al*, 1990; Schussler *et al*, 1992). Interestingly, in cancerous tissue, adenocarcinomas, such as human breast or colorectal carcinoma frequently show reduced CK8/18 expression, which correlates with tumour progression and poor outcome (Woelfle *et al*, 2004; Knosel *et al*, 2006). On the other hand, CK8/18 expression is upregulated in head and neck carcinoma (Xu *et al*, 1995; Gires *et al*, 2006), oral cavity carcinoma (Fillies *et al*, 2006), and transitional cell carcinoma of the urinary tract (Schaafsma *et al*, 1990; Southgate *et al*, 1999), and is associated with unfavourable prognosis (Fillies *et al*, 2006). *In vitro* analysis demonstrated the malignant role of CK8/18 expression, indicating that various CK8/18-overexpressing cell lines, such as human lung adenocarcinoma (Chu *et al*, 1997), human melanoma cells (Chu *et al*, 1996), and mouse L cells (Chu *et al*, 1993), have higher migratory/invasive abilities compared with the control.

Apart from CK8/18, earlier reports described loss of CK7 and acquisition of CK20 in colorectal carcinoma (Park *et al*, 2002), acquisition of CK7, and loss of CK20 in gastric carcinoma (Park *et al*, 2002; Kim *et al*, 2004), and acquisition of CK1, 5, 6, 8, 19 in squamous cell carcinoma (Xu *et al*, 1995; Chu and Weiss, 2002a, 2002b; Ikeda *et al*, 2008). Thus, it seems that CK expression in epithelial tissues can change with differentiation or malignant transformation (Hendrix *et al*, 1996) in an organ-specific manner (Kurokawa *et al*, 2006). Although earlier reports indicated upregulation of CK8/18 in OSCC during the pre-cancerous and cancerous stages (Grace *et al*, 1985; Lam *et al*, 1995; Takahashi *et al*, 1995; Viaene and Baert, 1995; Cintorino *et al*, 2001), to our knowledge, their role in prognosis has not been evaluated. Here, we assessed CK18 and CK8 expression levels in OSCC by using a series of 210 resected specimens. Furthermore, considering the clinical application of these CKs in the future, we also examined the expression levels of CK18 in biopsy specimens obtained by endoscopy.

## Patients and methods

### Patients and treatments

This retrospective study involved 210 patients with thoracic oesophageal cancer who underwent surgical resection in our hospital from 1998 to 2007. All 210 patients were confirmed to have OSCC by histopathological examination of endoscopic biopsy and considered suitable for surgery based on preoperative assessment. They included 23 females and 187 males, aged between 38 and 82 (median, 63.1 years). Table 1 lists patient characteristics. Of the total, 110 patients with cN1 received neo-adjuvant chemotherapy (NACT), which consisted of two courses of 5-fluorouracil (5-FU), cisplatin (CDDP), and adriamycin (ADM) (Akita *et al*, 2006; Yano *et al*, 2006; Matsuyama *et al*, 2007; Makino *et al*, 2008). Curative resection (R0), that is, oesophagectomy with two- or three-field lymphadenectomy, was performed in 199 patients (94.8%), whereas non-curative resection (R2) was carried out in the remaining 11 patients (5.2%); these patients were excluded from the survival analysis. None of the patients died of postoperative complications. Sixty-five patients with multiple metastatic lymph nodes in the surgical specimen received docetaxel or CDDP plus 5-FU postoperatively (Ando *et al*, 2003). In our hospital, patients with OSCCs that invade the airway or major blood vessels or accompanied by visceral metastasis are not indicated for surgery and therefore receive chemoradiotherapy or chemotherapy alone.

After surgery, the patients were surveyed every 3 months by physical examination and serum tumour markers, every 6 months by CT scan and abdominal ultrasonography, and every year by endoscopy until tumour recurrence was evident. Patients with tumour recurrence received chemotherapy or chemoradiotherapy, as long as their systemic condition permitted. The mean overall survival (OS) was 34.9 months and mean disease-free survival (DFS) was 22.2 months. The mean follow-up period after surgery was 38.8 months. This study was approved by the Human Ethics Review Committee of Osaka University School of Medicine and a signed consent form was obtained from each subject.

### Quantitative reverse transcription polymerase chain reaction analysis

Total RNA was extracted from fresh frozen tissue of resected tumours in 21 out of total 210 OSCCs using TRIzol Reagent (Invitrogen, Carlsbad, CA, USA). Complementary DNA (cDNA) was generated from 1 *μ*g RNA in a final volume of 20 *μ*l, containing oligo-(dT)_15_ primer, avian myeloblastosis virus transcriptase, with reverse transcription (RT) system (Promega, Madison, WI, USA). Polymerase chain reaction (PCR) analysis was performed by using LightCycler, a real-time monitoring thermal cycler. Polymerase chain reaction reaction mixture was prepared containing 2 *μ*l of cDNA template, 3 mmol l^−1^ MgCl_2_, 250 nmol l^−1^ of primer pairs, using LightCycler FastStart DNA Master SYBR Green I (Roche Diagnostics, Mannheim, Germany). The amount of each transcript was normalised against the expression of the housekeeping gene, glydecaldehyde-3-phosphate-dehydrogenase (GAPDH). Standard curve was constructed with 10-fold serial dilutions of cDNA obtained from non-cancerous oesophageal mucosal cell layers of tissue samples from 10 cases as a standard mixture. The sequences of PCR primers for GAPDH, CK18, and CK8 were as follows: forward primer 5′-CAACTACATGGTTTACATGTTC-3′, reverse primer 5′-GCCAGTGGACTCCACGAC-3′ used for amplification of GAPDH, forward primer 5′-ATCTTGGTGATGCCTTGGAC-3′, reverse primer 5′-CCTGCTTCTGCTGGCTTAAT-3′ for CK18, and forward primer 5′-TAGCACTGGGAACAGGAGA-3′, reverse primer 5′-TTTGACATTGGCAGAGCTA-3′ for CK8. The PCR cycling condition was set as follows: an initial denaturing step at 95°C for 10 min and 40 cycles at 95°C for 15 s, 58°C for 10 s, and 72°C for 25 s. The relative amount of cDNA in each sample was measured by interpolation on the standard curve, and then the relative ratio of CK18/GAPDH mRNA or CK8/GAPDH mRNA expression in log2 scale was calculated for each OSCC sample.

### Immunohistochemical analysis

The amounts of CK18 and CK8 proteins in the tissues were examined by immunohistochemical staining of formalin-fixed and paraffin-embedded serial sections. One representative slide with the deepest tumour invasion was selected from each patient and subjected to immunohistochemistry using the streptavidin–peroxidase method. Briefly, after deparaffinisation in xylene and dehydration in graded ethanol, endogenous peroxidase activity was blocked by incubation with 30 ml l^−1^ hydrogen peroxide for 20 min. Then tissue sections were heated for 40 min at 95°C in citrate buffer (0.05 mol l^−1^, pH 6.0) for antigen retrieval. After incubation with mouse monoclonal primary antibody DO10 (Bartek *et al*, 1991) (Novocastra, Newcastle, UK, dilution 1 : 40) for CK18 and TS1 (Johansson *et al*, 1999) (Novocastra, dilution 1 : 200) for CK8 for 14 h, the sections were stained by the labelled streptavidin biotin method. Negative controls of immunohistochemical reactions were performed by omitting the primary antibody. Positive staining of normal oesophageal gland at the non-cancerous area in the same section was used as an internal positive control. Furthermore, CK18 immunoreactivity was examined using the same antibody and methods in pretreated fiberscopic biopsy samples obtained from 83 of the 210 patients. The presence of CK18 or CK8 protein was judged ‘positive’ when the proportion of immunohistochemically stained cells was more than 50% of all observed cancer cells (Lam *et al*, 1995), or otherwise ‘negative’. All slides were assessed by two observers independently and then in conference in a blinded manner without any prior knowledge of the clinicopathological parameters. Characteristically, immunostaining of CK family of proteins yields clearly recognised tumour cells with sufficient intensity and frequency, with little or no background or non-specific staining. Therefore, the judgment of immunostaining was always consistent between the two observers and the results were the same on different cut-off lines around 10–50% of the proportion of immunohistochemically stained cells.

### Western blot analysis

Western blot analysis was performed to confirm the specificity of CK18 and CK8 antibodies (Miyata *et al*, 2001). Briefly, the protein extracted from tissue samples (20 *μ*g), MCF7 whole cell lysates (0.5 *μ*g) and recombinant protein of each CK18 and CK8 (ProSpec-Tany Technogene, Rehovot, Israel) (0.1 *μ*g) were separated using 7.5% polyacrylamide gel electrophoresis, followed by electroblotting onto a polyvinylidene difluoride membrane. The membrane was incubated with the primary antibodies at appropriate concentrations (anti-CK18 1 : 200 dilution, anti-CK8 1 : 500 for 12 h at 4°C, and anti-actin 1 : 2000 for 1 h at room temperature). Protein bands were detected using the Amersham-enhanced chemiluminescence detection system (Amersham Biosciences Corp., Piscataway, NJ, USA).

### Statistical analysis

Data are expressed as mean±s.d. Differences in continuous parameters between two groups classified by CK18 or CK8 protein expression were evaluated by the Mann–Whitney's *U* test. Correlations between CK18 expression on immunohistochemistry and various clinicopathological parameters were evaluated by the *χ*^2^ test and Fisher's exact probability test. Regression analysis was used to determine the correlation between CK18 and CK8 gene expressions using Pearson's correlation coefficient (*R* value). Prognostic variables were assessed by the log-lank test, and DFS and OS were analysed by the Kaplan–Meier test. Cox's proportional hazard regression model with stepwise comparison was used to analyse the independent prognostic factor(s). All statistical analyses were carried out using SPSS for Windows release 10 (SPSS, Inc, Chicago, IL, USA). A *P*-value of <0.05 was accepted as statistically significant.

## Results

### Cytokeratins 18 and 8 expression in oesophageal squamous cell carcinoma

**Figure 1:**
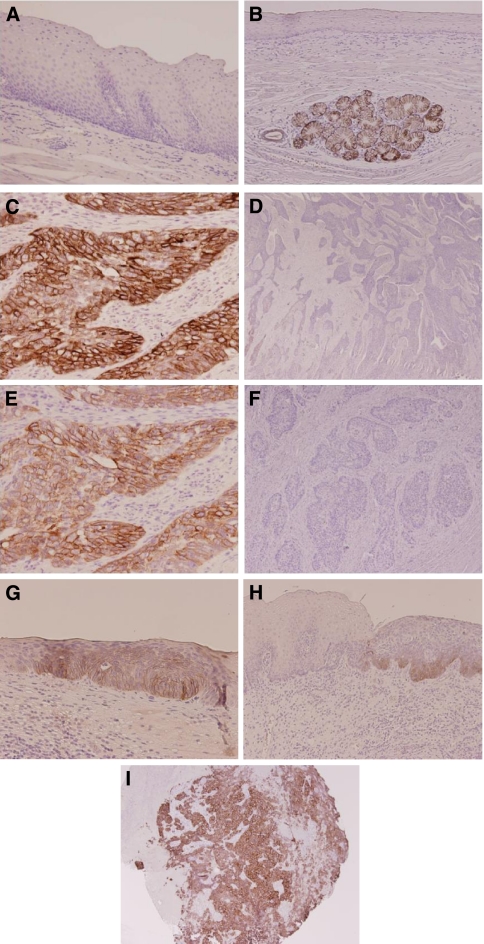
CK18/CK8 expression by immunohistochemical staining. (**A**) Normal squamous epithelium was negative for CK18 (magnification × 100). (**B**) Normal oesophageal glands showed positive staining for CK18, which was used as an internal control (magnification × 200). (**C**, **E**) Representative examples of CK18-positive (**C**) and CK8-positive (**E**) oesophageal squamous cell carcinomas, which showed staining in more than 10% of all tumour cells (magnification **C**, **E** × 200). (**D**, **F**) CK18-negative (**D**) and CK8-negative (**F**)- oesophageal squamous cell carcinomas showed almost no appreciable staining of tumour cells (magnification **D** × 20, **F** × 40). (**G**, **H**) CK18 (**G**) and CK8 (**H**) immunostaining in intra-epithelial neoplasia (magnification **G** × 200, **H** × 100). (**I**) CK18-positive oesophageal squamous cell carcinoma in a pretreatment biopsy specimen (magnification × 40).

Non-cancerous squamous epithelium showed no immunohistochemical staining for both CK18 and CK8, but adjacent proper oesophageal glands always showed strong immunostaining for both, which served as an internal positive control (Figure 1A and B). All 210 samples, including cancer and non-cancerous lesions in the same section, were evaluated for CK18 and CK8 protein expression by immunohistochemical analysis (IHC), respectively. Positive CK18 expression was identified in 90 (42.9%) cases, and the staining was mainly observed in the cytoplasm of tumour cells. The remaining 120 (57.1%) cases were negative (Figure 1C and D). Furthermore, 85 (40.5%) cases showed positive immunostaining for CK8 in the cytoplasm of tumour cells, whereas 125 (59.5%) were negative (Figure 1E and F). The positive staining for CK18/8 was almost homogeneous at single cancer nest and among different areas (surface, central, and deepest areas) of the cancer lesion. There was a significant correlation between CK18 and CK8 immunostaining (*P*<0.001); 69 (32.9%) patients showed positive immunostaining for both CK18 and CK8, with largely matching distribution, whereas 104 (49.5%) were negative for both. On the other hand, 37 (17.6%) patients showed discordant immunostaining, including CK18 positive and CK8 negative in 21 cases, and CK18 negative and CK8 positive in 16 (Table 2). Intra-epithelial neoplasia (dysplasia) was observed in 55 cases. Among them, CK18 and CK8 expressions were detected in 13 (23.6%) and 14 (25.5%) cases, respectively (Figure 1G and H).

**Figure 2:**
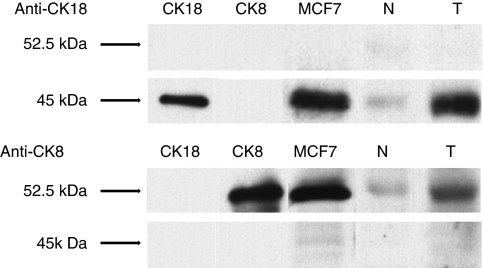
CK18/CK8 expression by western blot analysis. Each CK18 and CK8 expression was examined by western blot in oesophageal cancer tissue (T) and normal squamous epithelium (N) obtained from the same patient. Whole cell lysates of MCF7 and recombinant protein of each CK18 and CK8 were used as positive controls.

Western blot analysis demonstrated strong expression of CK18 and CK8, with molecular weights of 45 and 52.5 kDa, respectively, in whole cell lysates of MCF7 and recombinant proteins of CK18 and CK8. In cancer tissue obtained from one representative patient positive for both CK18 and CK8 expression by immunohistochemistry, CK18 and CK8 were strongly expressed, but not in normal squamous epithelial cells (Figure 2).

**Figure 3:**
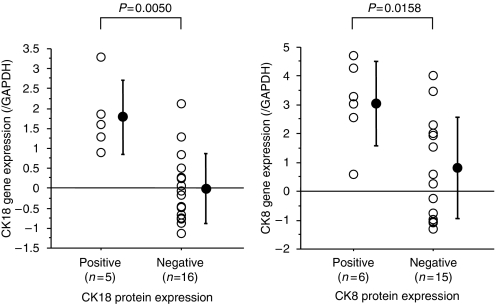
Relationship between protein expression and mRNA expression of CK18 and CK8. The relative ratio of CK18 mRNA expression in CK18-positive tumours (*n*=5) was significantly higher than in CK18-negative tumours (*n*=16). A similar trend was observed for CK8 mRNA expression in CK8-positive (*n*=6) and CK8-negative (*n*=15) tumours. Data are mean±s.d.

Quantitative RT–PCR analysis was performed in 21 representative cases to elucidate the mechanisms of gene transcription and the relationship between protein and mRNA expression levels of these molecules. It showed the expression level of CK18 mRNA in CK18-positive tumours (*n*=5) was significantly higher than that in CK18-negative cases (*n*=16) (1.78±0.92 *vs* −0.001±0.87, *P*=0.005). Similarly, the expression level of CK8 mRNA in CK8-positive tumours (*n*=6) was significantly higher than that in CK8-negative cases (*n*=15) (3.08±1.46 *vs* 0.84±1.75, *P*=0.016) (Figure 3). Regression analysis showed a significant correlation between CK18 and CK8 mRNA expression (*R*=0.822, *R*2=0.675, *P*<0.001).

### Correlation between cytokeratins 18 and 8 expression and clinicopathological parameters

Table 3 lists the correlations between CK18 and CK8 expression and various clinicopathological parameters. In comparison with CK18-negative OSCCs, the proportion of CK18-positive tumours was significantly higher among moderately poorly differentiated OSCCs (65.8% *vs* 92.2%, respectively, *P*<0.001), patients treated with NACT (45.0% *vs* 62.2%, respectively, *P*=0.018), advanced pathological T stage (pT3,4) (58.3% *vs* 73.3%, respectively, *P*=0.029), large number (⩾4) of pathologically positive lymph nodes (21.7% *vs* 44.4%, respectively, *P*=0.001), and advanced pathological stage (pStage III/IV) (45.0% *vs* 70.0%, respectively, *P*=0.0045). On the other hand, there were no significant correlations among CK18 expression and other parameters listed in Table 3, such as age, gender, tumour location, and clinical response to NACT.

Similarly, compared with CK8-negative tumours, the proportion of CK8-positive cases was significantly higher among moderately poorly differentiated OSCCs (65.6% *vs* 94.1%, respectively, *P*<0.001), patients treated with NACT (46.4% *vs* 61.2%, respectively, *P*=0.0485), and large number (⩾4) of pathologically positive lymph nodes (25.6% *vs* 40.0%, respectively, *P*=0.0340). On the other hand, there were no significant correlations among CK8 expression and other parameters listed in Table 3, such as age, gender, tumour location, pT, pStage, and clinical response to NACT.

### Survival rates

**Figure 4:**
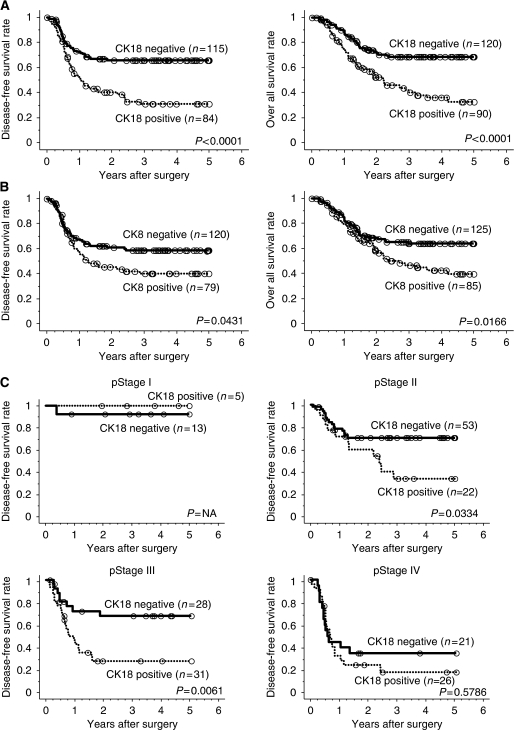
Survival rates according to CK18 and CK8 expression. (**A**, **B**) Disease-free survival curves (left, *n*=199) and overall survival curves (right, *n*=210) classified by CK18 (**A**) and CK8 (**B**) expression for all patients were plotted by Kaplan–Meier method. (**C**) Disease-free survival curves classified by CK18 expression in each pathological stage. Differences between the two groups were evaluated by the log-rank test.

Disease recurrence after curative resection was diagnosed in 90 (45.2%) of 199 patients with curative resection (R0) and the mean time to recurrence was 8.8 months. Deaths because of primary cancer occurred in 83 (39.5%) of the 210 patients and the mean time between surgical resection and death was 15.3 months. The 5-year DFS and OS rates for all patients were 51.6% and 54.0%, respectively. Patients with CK18-positive tumours had significantly poorer DFS and OS than those with CK18-negative tumours (5-year DFS: 30.6% *vs* 65.8%, respectively, *P*<0.001, 5-year OS: 32.8% *vs* 68.4%, respectively, *P*<0.001) (Figure 4A). CK8-positive tumours were also significantly associated with poorer DFS and OS than CK8-negative ones (5-year DFS: 40.0% *vs* 59.0%, respectively, *P*=0.043, 5-year OS: 39.6% *vs* 63.9%, respectively, *P*=0.017) (Figure 4B). There was significant prognostic difference in survival between CK18-positive and -negative groups according to pStage II (5-year DFS: 33.6% *vs* 70.6%, respectively, *P*=0.033) and pStage III (5-year DFS: 27.8% *vs* 67.9%, respectively, *P*=0.006), but not at pStage I (5-year DFS: 100% *vs* 92.3%, respectively, NS) or pStage IV (5-year DFS: 18.2% *vs* 35.0%, respectively, *P*=0.579) (Figure 4C).

Univariate analysis using Cox's proportional hazard model showed that the following parameters correlated significantly with DFS: pT stage, number of pathologically positive lymph nodes (pN number), lymphatic invasion (ly), venous invasion (v), CK18 expression, and CK8 expression (Table 4). Finally, the above parameters were entered into multivariate analysis. The results showed that pT, pN number, and CK18 expression significantly and independently influenced prognosis (i.e., DFS) (HR=1.909, *P*=0.020; HR=2.095, *P*=0.001; and HR=1.879, *P*=0.004, respectively, Table 4).

### Cytokeratin 18 expression in preoperative biopsy specimens

**Figure 5:**
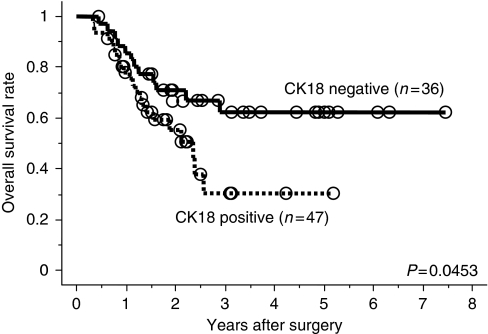
Survival rates according to CK18 expression in pretreatment biopsy samples. Overall survival curves (*n*=83) classified by CK18 expression were plotted by Kaplan–Meier method. Differences between the two groups were evaluated by the log-rank test.

In 83 patients, fiberscopic biopsy samples obtained before treatment, which included 2–5 tissue pieces in each patient, were also investigated for CK18 expression. Immunostaining for CK18 was basically similar among the obtained tissue pieces. Immunostaining showed CK18 expression in 47 (56.6%) cases (Figure 1I). The classification of CK18 immunostaining matched that of surgical specimens in 67 (80.7%) cases. Survival analysis also showed that pretreated CK18-positive tumours were also significantly associated with poorer OS than biopsy CK18-negative ones (5-year OS: 30.4% *vs* 62.2%, respectively, *P*=0.045) (Figure 5).

## Discussion

This study showed positive immunoreactivity to CK18 and CK8 in 42.9% and 40.5% of the surgical samples of OSCC, with a high concordance rate of IHC classification (82.4%). Positive CK18 and CK8 expression was significantly associated with pathological factors related to tumour progression (advanced pT, large pN number, and advanced pStage) and malignant phenotype (poor differentiation). CK18, in particular as a single marker, was identified as an independent prognostic predictor for DFS and OS and to influence prognosis especially in patients with pStage II/III tumours. The prognostic significance of CK18 expression was also confirmed in pretreatment biopsy samples, suggesting its potential suitability for clinical application in the future. To our knowledge, this is the first report that has identified CK18 and CK8 expression as a single predictor of progression and poor prognosis in OSCC patients after curative resection.

Cytokeratins are intermediate filaments and typical epithelial cell markers are expressed in organ-specific and differentiation-dependent manners (Kurokawa *et al*, 2006). Cytokeratin filaments are formed by tetrameric heteropolymers of two different types of CKs (types I and II) (Chu *et al*, 1993). With respect to their relation with cancer, the expression levels of some CKs, such as CK19, are abundant and stable among tumours, therefore these molecules are considered useful markers for micrometastases or speculation on occult primary cancer (Fujita *et al*, 2006; Liu *et al*, 2008). On the other hand, CK expression levels are reported to change dynamically with carcinogenesis, at least in some cases. Such changes include loss of CK7 and acquisition of CK20 in colorectal carcinoma (Park *et al*, 2002), acquisition of CK7 and loss of CK20 in gastric carcinoma (Park *et al*, 2002; Kim *et al*, 2004), or acquisition of CK 1, 5, 6, 8, 19 in squamous cell carcinoma (Xu *et al*, 1995; Chu and Weiss, 2002b; Ikeda *et al*, 2008). Furthermore, these changes correlated with malignant transformation or metastatic ability and were associated with poor prognosis (Kim *et al*, 2004). Also in OSCC, earlier reports indicated that CK expression profiles generally change along with malignant changes (Grace *et al*, 1985; Lam *et al*, 1995; Takahashi *et al*, 1995; Viaene and Baert, 1995; Chung *et al*, 2006), including induction of CK8, 14, 15, 17, 18, 19 expression (Grace *et al*, 1985; Lam *et al*, 1995; Takahashi *et al*, 1995) and loss of CK4, 5, 13 expression (Franke *et al*, 1981; Banks-Schlegel and Harris, 1984; Takahashi *et al*, 1995). Among the CKs, we evaluated CK8 and CK18 in this study.

Accumulation of CK8/18 proteins was observed in about 25% of the cases with dysplasia and half with OSCC, whereas normal squamous epithelium of oesophagus showed no such expression. Thus, CK8/18 expression is considered to be associated with tumour malignancy based on the finding of increased frequency of expression with tumour staging and correlation with unfavourable prognosis. A similar trend was also reported in other malignancies such as head and neck carcinoma (Gires *et al*, 2006), oral cavity carcinoma (Fillies *et al*, 2006), and transitional carcinoma of the urinary tract (Schaafsma *et al*, 1990). In contrast, some CK8/18-expressing adenocarcinomas show downregulation of such expression with progression, which correlates with poor outcome, as reported in human breast cancer (Takei *et al*, 1995; Schaller *et al*, 1996; Woelfle *et al*, 2004) and colorectal cancer (Knosel *et al*, 2006).

What are the functions of CK8/18 proteins? Although the exact roles of these proteins are unknown at this stage, there is evidence to suggest their involvement in invasive or growth properties of tumours (Chu *et al*, 1993; Raul *et al*, 2004) and drug resistance (Bauman *et al*, 1994; Ma *et al*, 2009). *In vitro* analysis using CK8/18 transfection technique showed conflicting results. In one study, mouse L cells transfected with CK8/18 showed enhanced migration and invasion abilities (Chu *et al*, 1993) whereas in another study transfection of CK18 gene in human breast cancer cells caused marked regression of malignancy (Buhler and Schaller, 2005). Another possibility of the functional roles of these proteins, CK8/18 expression levels may not directly correlate with malignant transformation, but change accompanying with other malignant signals, for example, epithelial–mesenchymal transition (Knosel *et al*, 2006). As recent evidence showed that oncogenes activating Ras signal-transduction pathways stimulate the expression of CK8 and CK18 genes through transcription factors, such as members of the AP1 (Jun, Fos) and ETS families (Oshima *et al*, 1996), the expression of CK8/18 may reflect integrated transcriptional activation of such transcription factors. In this study, there was a strong correlation between the expression of CK8 and CK18 with regard to both protein accumulation and mRNA level, which could indicate that CK8 and CK18 are regulated by some common signals. Further investigation is necessary to explore the regulatory mechanisms of CK8 and CK18 expression.

Survival analyses based on pathological stage by TNM classification (Sobin, 2002) were comparable to those of earlier reports of OSCC in Japan. In this study, CK18 had a significant prognostic value especially in patients with pStage II/III tumours, but not with pStage I/IV OSCCs. This finding suggests that the prognosis of patients with pStage II/III tumours is affected by malignant potentiality whereas that of pStage I/IV tumours is rather influenced by anatomical staging. Therefore, for the prediction of prognosis of patients with pStage II/III tumours, it might be useful to integrate CK18 expression level evaluation into pathological TNM classification.

With regard to the treatment strategies of OSCC patients, the use of CK18 expression and pathological TNM classification (Sobin, 2002) could be a valuable guide in decision making regarding adjuvant therapy. For example, postoperative chemotherapy might be useful for CK18-expressing pStage II/III tumours and pStage IV tumours regardless of CK18 expression, but not for pStage I tumours and CK18-negative pStage II/III tumours. Furthermore, because neo-adjuvant treatment has recently become the standard of care for patients with advanced OSCC, evaluation of pretreatment biopsy specimens is important. Our results indicated that though CK18 protein expression varied among OSCC, it was abundant and stable in each OSCC; oesophageal squamous cell epithelium is intrinsically negative for CK8/18 expression and almost all cancer tissue specimens positive for CK8/18 expression contained more than 50% immuno-positive cells relative to the total number of cancer cells, though in a few cases CK8/18-positive cells constituted only 10–50% of cancer cells. On the other hand, among CK8/18-positive cases, survival of patients with more frequent (more than 75%) expression of CK8/18 tended to show poorer prognosis but with no significant difference. Thus, our classification can be considered both practical and useful, and evaluation in biopsy specimen well represented the characteristic of the whole tumour. Usage of biopsy specimen would highly enhance the application of this molecule in clinical activity; as clinical staging can be performed precisely following recent advances in imaging modalities, it is possible that decisions regarding selection of neo-adjuvant therapy are based in the future on clinical TNM staging and CK18 expression level in pretreatment biopsy specimens. It should be noted that this study was based on analysis of squamous cell carcinoma, which is the dominant histopathological type in East Asian countries. Therefore, our results are not applicable to adenocarcinomas of the oesophagus, which is the major histopathological type in Western countries, which are characterised by overexpression of CK8 and CK18.

In conclusion, the results of this study showed that CK18 and CK8 expression as determined by IHC could be potentially a useful predictor of prognosis of OSCC patients after curative resection. These results may lead to the design of new treatment strategies for OSCC based on manipulation of CK18 and CK8 expression.

## Figures and Tables

**Table 1:** Patients characteristics (*n*=210)

Age (years)^a^	63.1 (38–82)
Gender (males/females)	187/23
Histopathology (well/mod/poor)	48/107/55
Location (upper/middle/lower)	27/101/82
Neo-adjuvant chemotherapy (yes/no)	110/100
cT (0/1/2/3/4)	0/28/63/94/25
pT (0/1/2/3/4)	0/39/35/120/16
cN (N0/N1/M1lym)	67/143/46
pN (N0/N1/M1lym)	61/149/51
Number of p (N0/1-3/4-7/8)	59/85/26/40
cStage (0/I/II/III/IV)	0/20/72/72/46
pStage (0/I/II/III/IV)	0/18/75/66/51

a Data are average and (range).

Well/mod/poor=well, moderately, and poorly differentiated squamous cell carcinoma; upper/middle/lower=middle, lower, and upper thoracic oesophagus.

cT, cN, cStage (clinical classification); pN, pT, pStage (pathological classification); and M1lym (distant lymph node metastasis) are based on TNM classification.

**Table 2:** Correlation between CK18 and CK8 protein accumulation examined by immunohistochemical staining

	**CK8**		
	**Positive**	**Negative**	**Total**
*CK18*			
Positive	69	21	90 (42.9%)
Negative	16	104	120 (57.1%)
			
Total	85 (40.5%)	125 (59.5%)	210 (100%)

*P*<0.0001 by Fisher's exact test.

**Table 3:** Correlation between CK18/CK8 and various clinicopathological parameters

	**CK18**			**CK8**		
	**Positive**	**Negative**	***P***	**Positive**	**Negative**	***P***
*Age*						
<65	55	61	0.1615	53	63	0.0922
>65	35	59		32	62	
						
*Gender*						
Male	83	104	0.2654	77	110	0.6555
Female	7	16		8	15	
						
*Histopathology*						
Well	7	41	<0.0001	5	43	<0.0001
Mod, poor	83	79		80	82	
						
*Location*						
Upper	13	11	0.2758	14	10	0.0766
Middle, lower	77	109		71	115	
						
*Neo-adjuvant chemotherapy*						
Present	56	54	0.0175	52	58	0.0485
Absent	34	66		33	67	
						
*pT*						
T0–2	24	50	0.0287	29	45	0.8831
T3–4	66	70		56	80	
						
*Number of pN*						
<4	50	94	0.0005	51	93	0.0340
>4	40	26		34	32	
						
*pStage*						
1	5	13	0.0045	8	10	0.6488
2	22	53		26	49	
3	36	30		29	37	
4	27	24		22	29	

Well, mod, poor=well, moderately, and poorly differentiated squamous cell carcinoma; upper, middle, lower=upper, middle, lower, and thoracic oesophagus.

pT, pN, pStage (pathological classification) based on TNM classification.

**Table 4:** Results of univariate and multivariate survival analyses of disease-free survival by Cox's proportional hazard model

	***n***	**HR**	**95%CI**	***P***
*Univariate survival analysis*				
Age (<65/⩾65)	109/90	1.097	0.722–1.667	0.6640
Gender (male/female)	178/21	1.522	0.703–3.292	0.2862
Histopathology (mod, poor/well)	152/47	1.664	0.955–2.900	0.0724
Location (middle, lower/upper)	179/20	1.004	0.520–1.938	0.9901
Neo-adjuvant chemotherapy (yes/no)	101/98	1.508	0.993–2.291	0.0542
pT (T3,4/T1,2)	125/74	2.781	1.689–4.579	<0.0001
Number of pN (⩾4/<4)	60/139	3.098	2.036–4.713	<0.0001
ly (present/absent)	161/38	3.074	1.486–6.357	0.0024
v (present/absent)	88/111	1.542	1.019–2.333	0.0405
CK18 expression (positive/negative)	84/115	2.388	1.565–3.643	<0.0001
CK8 expression (positive/negative)	79/120	1.528	1.010–2.313	0.0448
				
*Multivariate survival analysis*				
pT (T3,4/T1,2)	125/74	1.909	1.107–3.209	0.0199
Number of pN (⩾4/<4)	60/139	2.095	1.347–3.257	0.0010
ly (present/absent)	161/38	1.976	0.915–4.264	0.0829
v (present/absent)	88/111	1.023	0.655–1.599	0.9197
CK18 expression (positive/negative)	84/115	1.879	1.219–2.897	0.0043

For abbreviations, see Tables 1, 2 and 3; ly=lymphatic invasion; v=venous invasion; HR=hazard ratio; 95% CI=95% confidence interval.
